# An ovalbumin fusion strategy to increase recombinant protein secretion in chicken eggs

**DOI:** 10.1186/s13036-023-00390-4

**Published:** 2024-01-11

**Authors:** Long Xie, Zhenwen Huang, Meiyu Lan, Yaqi Cao, Lingling Sun, Lang Zhang, Erwei Zuo, Yangqing Lu

**Affiliations:** 1 State Key Laboratory for Conservation and Utilization of Subtropical Agro-bioresources, Guangxi University, Nanning, China; 2 Shenzhen Branch, Guangdong Laboratory for Lingnan Modern Agriculture, Key Laboratory of Synthetic Biology, Ministry of Agriculture and Rural Affairs, Agricultural Genomics Institute at Shenzhen, Chinese Academy of Agricultural Sciences, Shenzhen, China

**Keywords:** Genetically modified chicken, Deposited foreign protein in eggs, Recombinant protein secretion, Nonsecretory protein secretion, Bioreactor

## Abstract

Maternal secretion of recombinant proteins into chicken eggs may provide a viable approach for pharmaceutical production but remains limited by poor secretion efficiency through the membrane of oviduct cells, despite high expression levels. Here, we used site-specific integration of an EGFP fused to the OVAL gene by a rigid linker, (EAAAK)_3_, at the endogenous ovalbumin locus in chicken primordial germ cells to generate OVAL-E3-EGFP transgenic chickens, with transgenic chickens expressing CMV immediate enhancer/β-actin-driven EGFP (CAG-EGFP) as a non-secreted control. In OVAL-E3-EGFP chickens, EGFP protein produced in maternal oviducts accumulates to high levels in eggs, but not in eggs of CAG-EGFP chickens. These results indicated that the secretion of foreign proteins can be substantially increased through fusion to the highly secreted endogenous ovalbumin. This study describes a basis for high yield recombinant protein expression in chicken eggs, enabling rapid and scalable production of numerous pharmaceutical proteins or metabolites.

**Supplementary Information:**

The online version contains supplementary material available at 10.1186/s13036-023-00390-4.

## Introduction

Increasing global demand for recombinant pharmaceutical proteins has resulted in significant research focus on the development of alternative production platforms, including the use of transgenic animals. At present, the majority of recombinant proteins are generated and purified from prokaryotes and are thus accompanied by several problems such as residual bacterial endotoxin lipopolysaccharide (LPS) [1]. Proteins derived from animal cell such as CHO [2] have overcome these issues, but there still exist a series of problems regarding yield and cost. In contrast, recombinant proteins derived from animal products, such as eggs, may be produced at high yields with fewer unpredictable effects [3]. In particular, chicken may provide several advantages over mammalian systems, including shorter time-scale for set up and scalability in genetically modified flocks, in addition to lower costs and reduced immunogenicity of the purified product [4].

The first reported transgenic hens expressing a foreign protein were generated using a replication-deficient retroviral vector based on avian leukosis virus (ALV) with a randomly inserted β-Lactamases gene under the control of a cytomegalovirus immediate-early (CMV) promoter. However, transgene expression was relatively low using this vector construct and resulted in poor yields of recombinant protein in eggs [5]. Later studies incorporating the upstream or downstream region of the Ovalbumin gene led to higher expression levels of foreign proteins such as mAbF1 [6], humanized ScFv-Fc mini-antibody, and IFNβ1a [3, 7] in oviduct cells, although secretion into eggs remained inefficient or limited in proteins with secreting capability. To address this issue, secretion signal peptides, such as a sequence encoding the lysozyme signal peptide [8], were proposed to enhance the secretion of exogenously expressed proteins into the egg. However, the secretion of the recombinant fusion protein can still be limited by differences in the strength of signal peptides [9], and fusion of different recombinant proteins might interfere with the signal peptide function, reducing the efficiency of secretion.

Here, we describe a foreign-signal-peptide-free method for high-yield production of exogenous proteins in chicken eggs by exploiting the high-efficiency secretion of endogenous ovalbumin protein from chicken oviducts into eggs. We used CRISPR-CAS9-mediated site-specific integration of an EGFP reporter fused to the ovalbumin CDS with a rigid (EAAAK)_3_ linker at genomic ovalbumin locus in primary germline cells (PGCs) via homology-mediated end joining (HMEJ) [10]. We then measured the efficiency of reporter secretion in eggs. In addition, a mCherry gene was integrated near the ovalbumin locus without a secreted fusion partner, and transgenic chickens expressing non-secreted, CMV-driven EGFP were generated by the piggyBac transposase, which together served as negative controls for secretion. This study describes a process for genetic modification of chickens that enables high production and secretion of recombinant proteins.

## Result

**Fig. 1:**
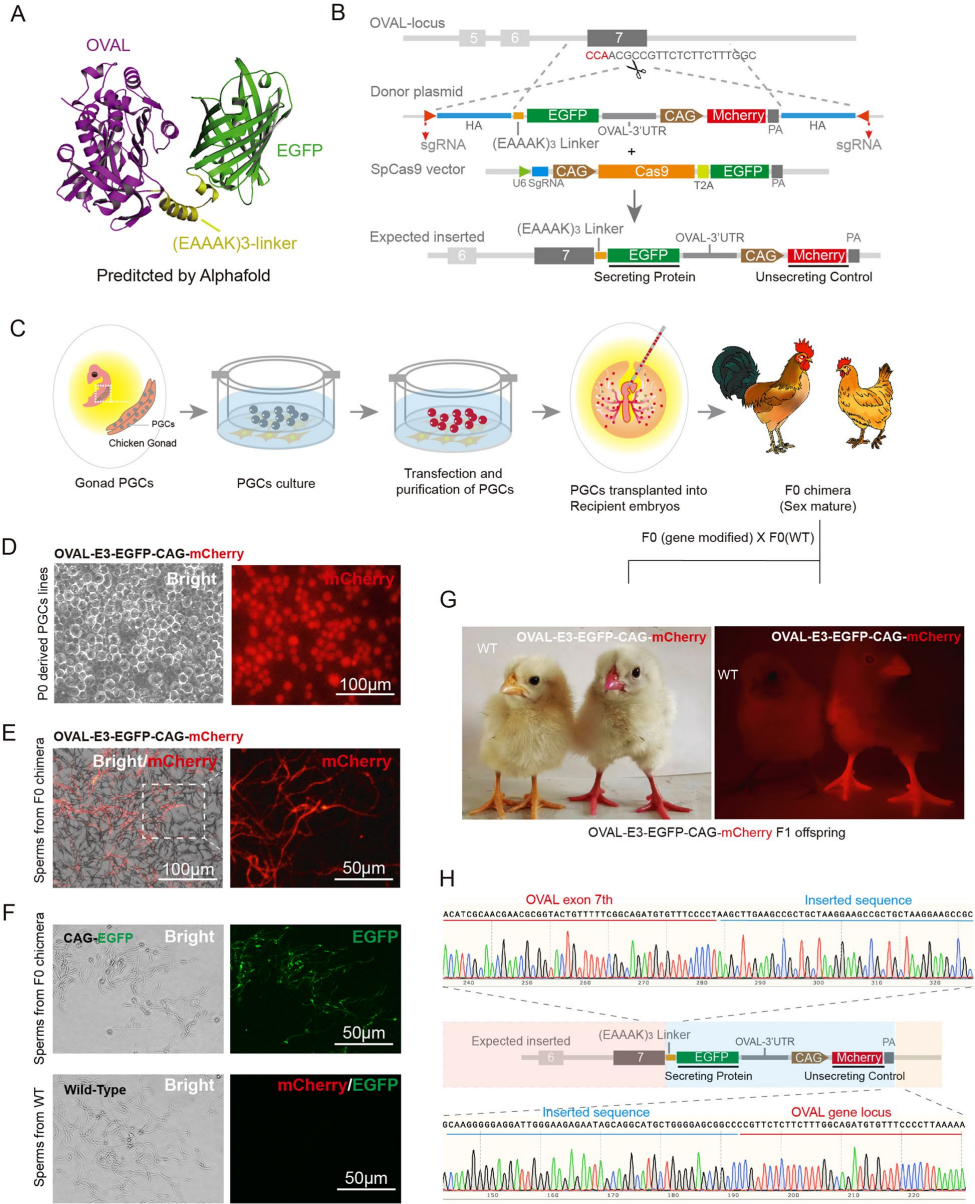
The generation of OVAL-E3-EGFP chickens. **A** Ovalbumin-(EAAAK)_3_-EGFP protein 3D structure predict by alphafold. **B** strategy of site-specific gene integration in ovalbumin locus. **C** schedule for generating the OVAL-E3-EGFP off-spring. **D** OVAL-E3-EGFP PGCs cell line established by FACs. **E** The sperms fluorescence was presenting in the sperms of OVAL-E3-EGFP recipients. **F** EGFP fluorescence was detected in the sperms of CAG-EGFP recipients but not in wild-type group. **G** OVAL-E3-EGFP offspring exhibited a distinct and robust mCherry signal in their comb, beak, skin, and claw. **H** Sanger sequencing reveals the precise integration of linker-EGFP sequence in ovalbumin locus

Prior to the fusion of exogenous proteins, it was first necessary to identify a linker peptide that sufficiently separated the functional domain of ovalbumin from the transgenic protein in order to avoid the effects of protein-protein interactions (PPI). To this end, alphafold [11] was used to predict the OVAL-linker-EGFP protein structure for several candidate linkers including three flexible fusion linkers, GS, (GS)_3_, and 32aa (Fig. S1A, B and C), and two rigid linkers, (EAAAK)_3_ and (EAAAK)_5_ (Fig. 1A and Fig. S1D). Among the coupled structures predicted for GS, (GS)3, and 32aa, ovalbumin and EGFP appeared to more readily undergo PPI even after extending linker length to 32aa (Fig. S1C, D and E), whereas, EGFP was more likely to remain separated from OVAL in both (EAAAK)_3_ and (EAAAK)_5_ predicted structures (Fig. S1A and B). These results suggested that the (EAAAK)_3_ and (EAAAK)_5_ rigid linkers could potentially avoid the effects of PPI between ovalbumin and EGFP (Fig. 1A). Based on its smaller size, we selected the (EAAAK)_3_ linker for subsequent experiments.

Subsequently, we adopted a strategy known as homology-mediated end joining (HMEJ) to achieve site-specific integration at the ovalbumin gene locus (Fig. 1B, S2A) in chicken cells. This HMEJ strategy has been reported as a highly efficient method for precisely integrating in chicken cells [10]. To begin with, we initially constructed two plasmids: an all-in-one CRISPR/Cas9 plasmid and an integrating donor plasmid. Within the integrated-sequence (between HAL and HAR) of donor plasmid, we fused the EGFP gene to the 3′ end of the ovalbumin coding sequence (CDS) using a (EAAAK)_3_ linker (E_3_ linker). Furthermore, as a negative control for co-secretion along with OVAL, we inserted the mCherry gene driven by the CAG promoter into the donor plasmid. Once integrated into the chicken genome, this mCherry gene will be expressed independently (Fig. 1B).

After the plasmid construction, the donor plasmid carrying these DNA elements was co-transfected into primary germline cells (PGCs) freshly isolated from chicken gonads along with the CRISPR/Cas9 system plasmids (Fig. 1C). At 72 hours post-transfection, the mCherry-positive cells were isolated by fluorescence-activated cell sorting (FACS) and transferred to fresh medium for further proliferation. At 14 days post-transfection, approximately 5–10 × 10^3^ PGCs were transplanted into the vascular system of 54-hour-old chicken embryos. The transgenic chicks were hatched and raised to sexual maturity, then mated with wild-type (WT) domestic three-yellow hens to generate F1 offspring (Fig. 1C). In addition, a transgenic chicken group expressing EGFP generated via transposase system served a second control for EGFP protein secretion (Fig. S2B). To generate these chickens, a piggyBac transposon plasmid carrying the EGFP gene controlled by the CAG promoter and a plasmid containing the transposase were co-transfected into PGCs (as CAG-EGFP group), enriched by FACS, then expanded in culture and transplanted using the same method as for the CRISPR/Cas9-edited chickens.

Fluorescence detection of mCherry+ cells derived from the gonads revealed abundant expression in the OVAL-E_3_-EGFP group F1 embryos (Fig. 1D) at embryonic day 7 (E7), suggesting that germline transmission of donor PGCs was successful. At sexual maturity, sperm was collected from recipient chickens and fluorescence microscopy confirmed the prevalence of mCherry+ sperms (Fig. 1E). Moreover, the hatched OVAL-E3-EGFP offspring exhibited a distinct and robust mCherry signal in their comb, beak, skin, and claw (Fig. 1G). Through Sanger sequencing analysis of the ovalbumin gene region, we confirmed the precise integration of the E_3_-linker-EGFP sequence at the 3′ end of the 7th exon in these offspring (Fig. 1H). Furthermore, we conducted an in-depth analysis of the potential off-target effects on the top 20 predicted off-target sites identified by Casoffinder [12]. Our investigation revealed no evidence of editing or integration at these specific genomic loci (Table. S3). In the CAG-EGFP group, we also observed the presence of EGFP protein in both primordial germ cells (PGCs) (Fig. S3) and sperms (Fig. 1F). The hens from CAG-EGFP group were rised into sex mature to laid eggs as the control of OVAL-E3-EGFP group.

**Fig. 2:**
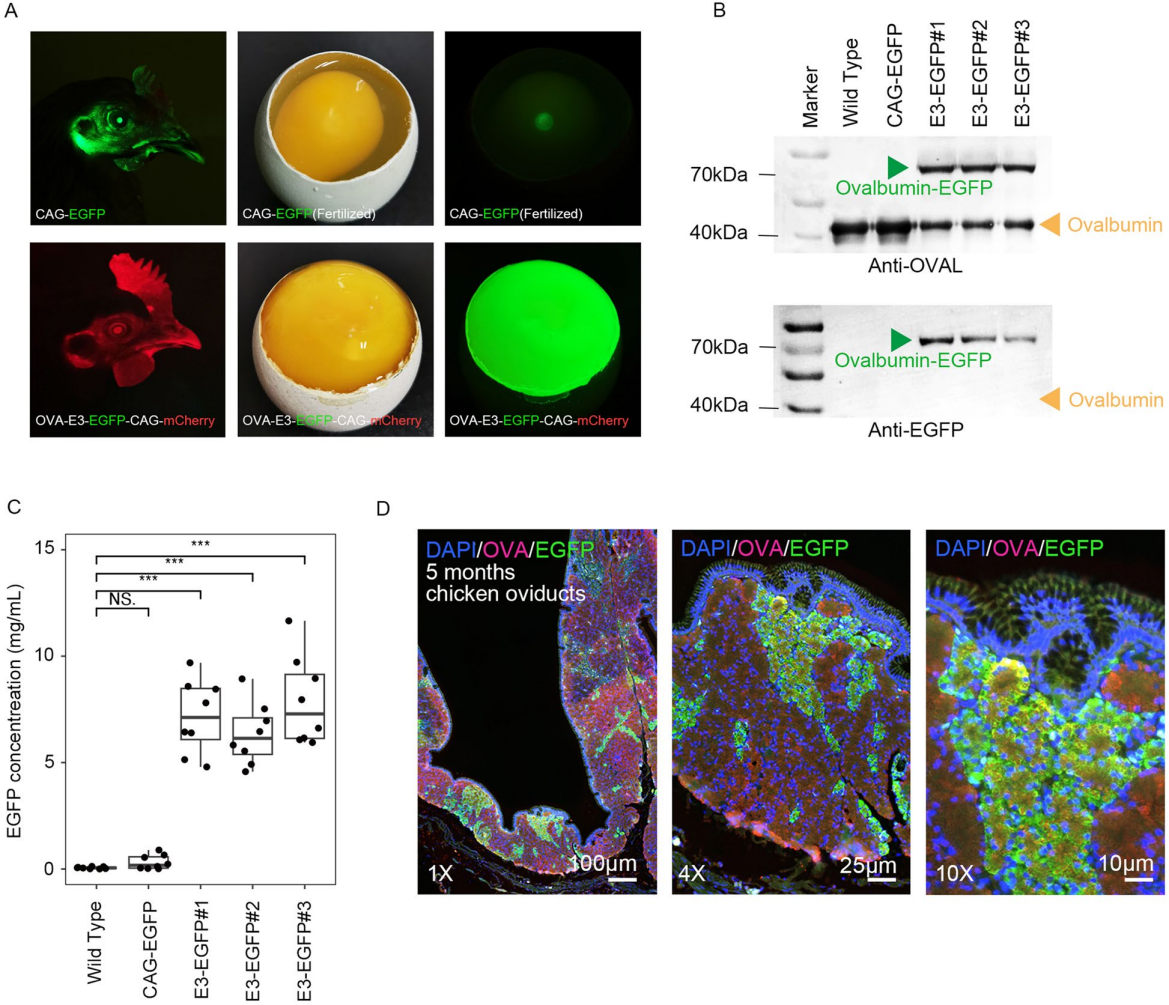
Ovalbumin fusing strategy increases the high yield secreting of foreign EGFP protein in chicken eggs. **A** The EGFP fluorescence was detected in F1 OVAL-E3-EGFP chicken eggs. **B** Western blot of egg white from different groups suggested that EGFP protein was fused to ovalbumin protein and secreted into eggs with a high proportion. **C** The EGFP protein concentration in egg white from different groups. **D** The dissection of OVAL-E3-EGFP chicken oviducts with immune staining with antibodies of ovalbumin and EGFP

At 5 months old, eggs were collected from OVAL-E3-EGFP chickens for detection of recombinant protein secretion. In this eggs, we have observed distinct volume differences (lengthwise and crosswise) between OVAL-E3-EGFP eggs and ordinary eggs, as well as a significant reduction in the proportion of egg white volume (*p-*value = 0.00016; OVAL-E3-EGFP: 23.69 ± 0.907 g per egg, Wild-type: 28.06 ± 1.569 g per egg) (Table. S3). These findings suggest that the expression of the endogenous ovalbumin gene has been markedly affected following EGFP insertion. In the unfertilized eggs of OVAL-E3-EGFP chickens, we observed robust EGFP fluorescence throughout the egg white, whereas the EGFP signal was restricted to the ovule in fertilized CAG-EGFP eggs (Fig. 2A), suggesting that the exogenous OVAL-(EAAAK)_3_-EGFP fusion protein was indeed successfully secreted into eggs from the maternal oviduct epithelial cells, but not in the CAG-EGFP eggs. Western blots confirmed that the egg whites of OVAL-E3-EGFP chickens contained high levels of EGFP with ovalbumin (Fig. 2B), which accounted for 43.3% ± 6.1% of total ovalbumin protein band density, accumulating to concentrations close to 6.33 ± 1.37 and 7.76 ± 2.1 mg/mL in egg white (Fig. 2C). At the same time, mCherry protein was undetectable in these eggs by fluorescence microscopy (Fig. S4), supporting that fusion with OVAL was necessary for secretion of the reporter protein, regardless of robust expression levels. Observation of oviduct tissue sections from OVAL-E3-EGFP chickens revealed that although EGFP expression was abundant in this oviduct tissue, not all oviduct epithelial cells expressed EGFP protein (Fig. 2D, S5), whereas almost all oviduct cells from CAG-EGFP chickens expressed EGFP protein (Fig. S6). Interestingly, despite the lack of EGFP expression, ovalbumin protein remained abundant in the EGFP- cells in oviducts (Fig. 2D). This phenomenon indicated that an event occurred during the EGFP protein expression process in this EGFP- cells.

**Fig. 3:**
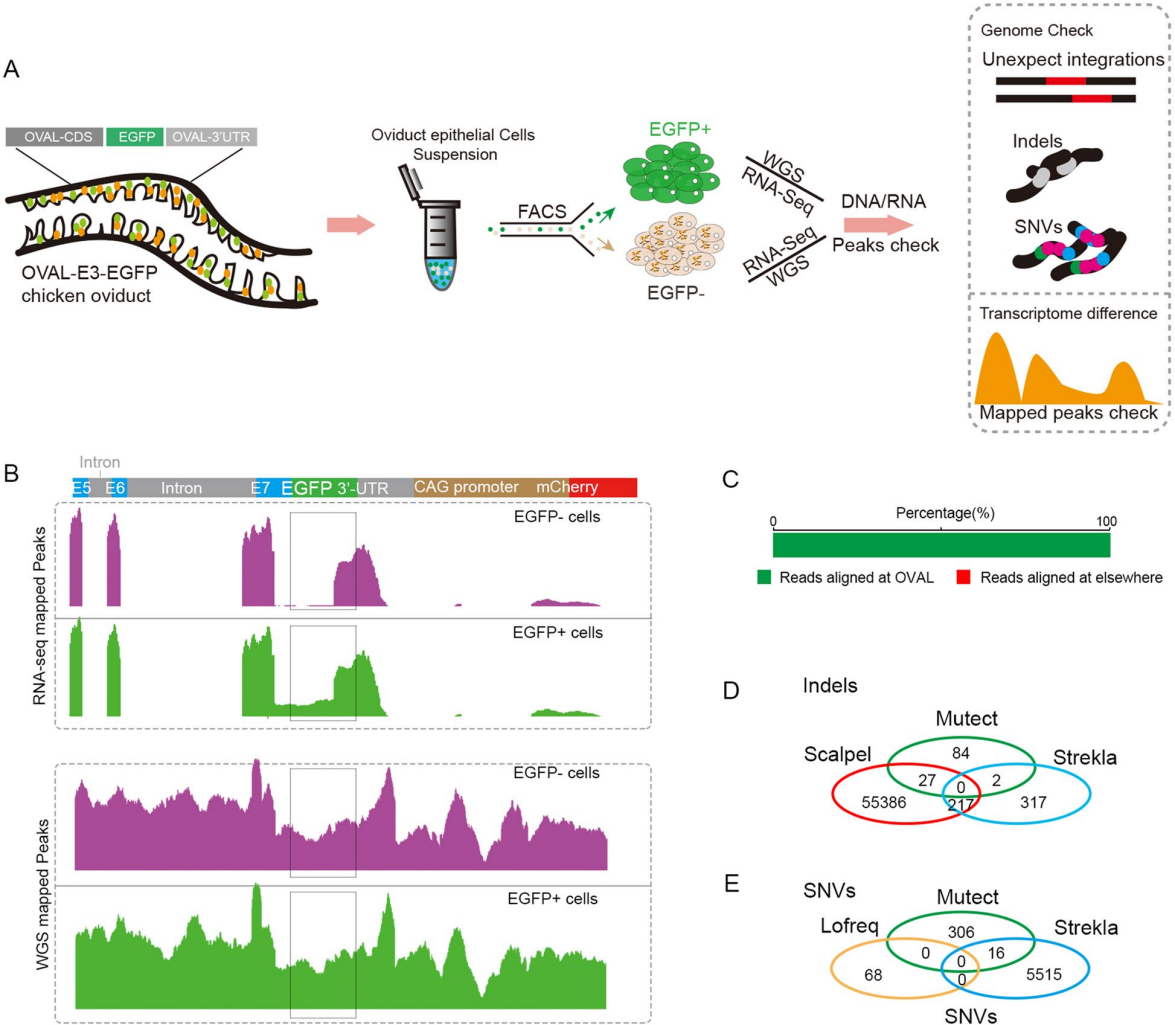
Genomic and transcriptomic analysis of EGFP+/EGFP- cells from OVAL-E3-EGFP chicken oviducts. **A** Strategy of detecting the genome mutations and RNA expression difference between EGFP+ and EGFP- cells from oviduct epithelial cells of OVAL-E3-EGFP chickens. **B** RNA-seq mapped peaks and WGS mapped peaks around the ovalbumin locus. **C**, **D** Indels (F) and SNVs(G) analysis of EGFP- epithelial cells of OVAL-E3-EGFP chicken oviduct

To validate the accurate insertion of the EGFP foreign fragment at the albumin locus and examine the possibility of off-target integrations at other loci, we then respectively conducted whole genome sequencing (WGS) with 55x coverage in the EGFP+ and EGFP- cells from oviduct to identify potential genome integrations or mutations (Fig. 3A, S7). Analysis of WGS data showed that reads for EGFP mapping to sequence around the insertion site in the ovalbumin locus were aligned (Fig. 3B), and no unexpected integration events occurred in other genome loci (Fig. 3C), suggesting that he EGFP gene was correctly fused to the 3′ end of the ovalbumin CDS in both EGFP+ and EGFP- cells. Analysis of genome-wide insertions or deletions (indels) and single nucleotide variants (SNVs) using three separate variant calling algorithms, with overlapping loci considered bona fide variants, showed no overlap in either indels (Fig. 3D) or SNVs (Fig. 3E), indicating that no genomic differences related to EGFP expression were detectable EGFP+ and EGFP- cells.

To further substantiate if the expression of EGFP had been silenced at the RNA transcription step, we conducted RNA sequencing (RNA-seq) and isolated reads that mapped to the EGFP insertion site at the ovalbumin locus of both EGFP-positive and EGFP-negative cells (as depicted in Fig. 3A). Aligning these reads for the EGFP gene showed that they were expressed at similar levels as the ovalbumin locus in EGFP+ cells, whereas markedly fewer EGFP gene reads than OVAL reads were detected in EGFP- cells (Fig. 3B). Taken together, these results showed that no genome mutations responsible for suppressing EGFP expression could be detected in cells lacking EGFP, whereas RNA expression was obviously diminished in EGFP- oviduct cells of OVAL-E3-EGFP chicken. Further research is needed to investigate the causes of these phenomena.

## Discussion

Currently, recombinant pharmaceutical proteins comprise an essential portion of human clinical therapies. As germline transmission methods have been well established in chicken [13], exogenous protein expression in chicken eggs has been considered a feasible potential strategy for high-yield therapeutic production. Previous studies have shown that the use of transcriptional regulatory elements, such as sequence around the ovalbumin CDS locus, can increase the expression levels of foreign proteins, but the secretion of these proteins is limited [3].

The utilization of endogenous gene loci has significantly enhanced the production yield of exogenous proteins. In a study by Oishi et al. [6], a fragment of the human interferon beta (hIFNb) gene, which codes for a secretory protein, was ingeniously inserted into the 2nd exon of the ovalbumin gene locus. This approach capitalizes on the naturally high levels of ovalbumin protein expression in chicken oviducts and utilizes specific amino acid residues within the ovalbumin gene to facilitate translocation of the exogenous protein across the oviduct cell membrane. The study successfully achieved an impressive exogenous protein production level of 3.5 mg/ml, highlighting the potential of utilizing the ovalbumin gene for protein production in chicken eggs.

In our research, we fused a non-secretory protein, EGFP, to the CDS end of ovalbumin. By utilizing the full-length ovalbumin protein and related regulatory elements, including 3′-UTR, we achieved a protein production level of 6.33 ± 1.37 ~ 7.76 ± 2.1 mg/mL in egg white. This suggests that the EAAAK3-fused strategy is an effective method for producing non-secretory proteins in eggs. Furthermore, this production efficiency allows for the incorporation of various cleavage tags, such as His, Flag, and others, between these two components. This capability holds great promise for future industrial-scale production of therapeutic proteins.

By contrast, the EGFP reporter was undetectable in egg whites of the CAG-EGFP transgenic control group in our study. Additionally, insertion of the mCherry protein, alone, around the ovalbumin locus resulted in no detectable accumulation of this reporter in the egg white of OVAL-E3-EGFP chickens. These results supported that transgene expression levels are independent of their secretion in chickens, which is consistent with findings in the previous studies [3, 5].

To improve secretion of the EGFP transgene, its coding sequence could be directly fused with that of ovalbumin protein by a linker, which we found resulted in both high protein expression and secretion. It warrants mention that the EGFP expressed in this work was 239aa, considerably larger than many recombinant pharmaceutical proteins, such as hEGF and b-IFN, suggesting that this strategy may be suitable for production of many non-secreted proteins. In *G.gallus* eggs, ovalbumin accounts for approximately 54% of the total protein content [3], suggesting that a transgene fused to ovalbumin could potentially accumulate at similar, if not equivalent, levels. The OVAL-E3-EGFP chickens described here were generated by hybridization and were therefore heterozygous for the modified ovalbumin locus, but still accumulated substantial quantities of EGFP in eggs. Interestingly, not all oviduct epithelial cells in the OVAL-E3-EGFP transgenic chickens expressed high levels of EGFP, although no relevant mutations could be detected. In future work, we will examine possible epigenetic or post-transcriptional regulatory mechanisms that might lead to this heterogeneous pattern of expression. This study ultimately provides a simple, robust method for high yield production of commercially valuable, non-secreted, recombinant proteins in the eggs of genetically modified chickens.

## Material and methods

### Alphafold prediction

The protein structure prediction was performed in the alphafold2 software under the default arguments by inputting the full-length amino acid sequence of chicken ovalbumin-coupled EGFP proteins with different linkers.

### Animal care

The use and care of animals complied with the guideline of the Biomedical Research Ethics Committee of the State Key Laboratory for Conservation and Utilization of Subtropical Agro-bioresources (in Guangxi University, Nanning, China).

### Plasmid construction

The CRISPR/Cas9 plasmid targeting ovalbumin was constructed base on the plasmid PX330 (PX330 was a gift from Feng Zhang; Addgene plasmid #42230). Briefly, we fused an EGFP after the coding region of Cas9 protein to construct a U6-sgRNA-CAG-Cas9-EGFP plasmid, linear the plasmid by BbsI (New England Biolab, NEB) digestion and inserted the annealed sgRNA oligos by T4 ligation (NEB). For donor plasmid, 800 bp DNA sequences around sgRNA targeted sites were cloned from the genomic of three-yellow-chicken and fused to the sides by EGFP-3′-UTR-CAG-mCherry fragments by NEBuilder® HiFi DNA Assembly Master Mix (NEB). The piggyBac transposon plasmid containing CAG-EGFP elements and transposase plasmid was a gift from Kehuan Lu professor at Guangxi university. All these plasmids were extracted following the manuals of E.Z.N.A Endo-free plasmid mini kit.

### PGCs deriving

For primary PGCs deriving, fertilized eggs were collected from Donglan chickens, a black feather chicken breed native to Guangxi Province, China. Embryonic gonads were isolated from 7-day-old chicken embryos (stage HH27–31), trypsinized with 0.05% trypsin-EDTA at 37 °C for 10 min, neutralized in DMEM/F12 supplemented with 10% fetal bovine serum FBS and plated in 24-wells plates with 24-well transparent membrane (PET) inserts (1.0 μm; Millipore, Stafford, VA, USA). After 4–5 h incubation in 5% CO2 at 37 °C, suspended cells were collected by gentle pipetting transferred into PGCs culture medium coupled with an insert (mKO-Insert), and kept passaged or changed medium every 2 days. PGCs culture medium was based on a Knockout DMEM (osmolality:250 mOsmol/kg, customized in Thermo-Fisher) supplied with 0.2% chicken serum, 1% fetal bovine serum (FBS; Hyclone), 1 X NEAA, 0.1 mM of b-mercaptoethanol, 4 ng/mL human recombinant FGF(R&D), 1.2 mM sodium pyruvate, 1 X GS nucleoside (Milipores), 100 μg/mL sodium heparin (Sigma), 25 ng/mL activin A (PeproTech), and 1 X B27 supplement. Without the specific annotation, the reagents mentioned above were purchased from Thermofisher.

### Cells transfection, FACs, and transplant into recipient embryos

For PGCs transfection, we used Lipofectamine3000 Reagent according to the manufacturer’s instructions. A total of 2 μg of plasmids (Cas9: donor = 1: 1) were used for each well of a 24-wells plate, and the transfection solution was removed 12 hours later. mCherry positive (mCherry+) cells were isolated by FACS 3 days after transfection and purified again by second FACS 4–6 days later. mCherry+ cells were cultured in the mKO-insert system and proliferated for transplantation into recipient embryos. For recipient embryo injection, 5–10 × 10^3^ PGCs were injected into the vascular system of 53-55 h recipient embryos.

### Germline transmission and chicken lines keeping of OVAL-E3-EGFP chickens

After the recipient chicken hatched, they were raised into sex matured. The sperms from recipient cocks were collected for fluorescence observation to confirm the germline transmission of mCherry+ PGCs. Recipient chickens were made with wild-type three-yellow chicken, and F1 offspring were confirmed by fluorescence detection. For OVAL-E3-EGFP chicken lines keeping, the male chickens were kept for generating offspring and female chickens were used for foreign protein production.

### Immunofluorescence

The immunofluorescence (IF) staining was performed based on the paraffin dissection staining method. The oviducts from different chickens were freshly collected and overnight fixed with 4% paraformaldehyde (PFA), washed with PBS and dehydrated in a gradient series of alcohol solutions, and embedded in paraffin. Tissue in paraffin was then sliced up to sections (5 μm), de-paraffinized, and rehydrated for IF analysis. For IF analysis, the monoclonal antibody against EGFP (Abcam, ab184601) and Ovalbumin (Abcam, ab306591) were used as 1st antibody. The second antibody with Alexa Flour 488 label or Alexa Flour 568 (Thermofisher) was used to visualize the expressed protein in sections. For cell nuclear staining, DAPI with Alexa Fluor 405 (Thermofisher) was used.

### Eggs fluorescence detection and analysis

EGFP concentration in eggs was measured according to the standard curve established based on the EGFP protein Standard sample. For the EGFP concentration standard curve established, the EGFP protein standard (Beyotime, P7410) was purchased and diluted into different concentrations as standard samples. Two μL of EGFP samples in different concentrations were then moved to the surface of a slice, covered by cover slide, taken photos under the fluorescence microscope by the same exposing time (Fig. S7), and analyzed by the ImageJ software. For EGFP concentration evaluation, the eggs from wild-type chickens, CAG-EGFP chickens, and OVAL-E3-EGFP chickens were respectively collected for fluorescence detection and analysis. The thick albumen and thin albumen were both extracted from the eggs and mixed well by carefully pipetting, and 1 mL of egg white mixture was 1:30 diluted into PBS as the sample for further analysis. The same density analysis protocol as EGFP standards was used to get the fluorescence density of egg white samples, and concentrations were calculated by using the formula of the standard curve.

### Western blot analysis

Egg white from different chickens was collected and 1:1000 diluted into PBS and their concentration was detected by bicinchoninic acid (BCA) assay reagent (Beyotime Biotechnology). Sodium dodecyl sulfate-polyacrylamide gel electrophoresis (SDS-PAGE) sample buffer was then mixed with samples and boiled for 5 min and equal amounts of proteins (20-40 μg) were electrophoresed on SDS-PAGE and transferred to polyvinylidene difluoride (PVDF) membranes (Bio-Rad). For first antibodies incubation, the monoclonal antibody against EGFP (Abcam, ab184601) and Ovalbumin (Abcam, ab306591) was used, and PVDF membranes were overnight incubated in 4 °C with 1st antibodies. 2nd antibodies labeled with alkaline ahosphatase (Beyotime) and BCIP/NBT Alkaline Phosphatase Color Development Kit (Beyotime) were used for visualization of EGFP protein or ovalbumin protein bands. The band grey density assays were performed by using ImageJ.

### Oviduct EGFP+/− cells WGS and RNA-seq analysis

For cell isolation, fresh oviducts from OVAL-E3-EGFP chickens were dissected and washed three times in PBS supplied with 5x Anti-Anti (Thermofihser). The tissue on the surface of the oviduct inner wall was collected and trypsinized by 0.25% enzyme for 10 minutes at 37 °C, the suspension was then put through a 70 μm cell strainers, and flowed cells were collected and re-suspended by DMEM medium supplied with 2% of FBS. Freshly isolated cells were separated into EGFP+ and EGFP- groups by FACS.

For WGS analysis, the genomic DNA of EGFP+/EGFP- cells was extracted by using the DNeasy blood and tissue kit (Qiagen) according to the manufacturer’s instructions. WGS was performed at mean coverages of 55x by BGI DNBSEQ-T7. The qualified sequencing reads in WGS data were mapped to the reference genome (GRCg7b) by using BWA (v0.7.17), and Picard tools (v2.25.7) were then used to sort and mark duplicates of the mapped BAM files. To save computing time and resources, Strelka (v2.9.10) was first run for the detection of whole genome de novo Indels and SNVs. Then 200 bp upstream and downstream of the mutation location were selected as candidate regions. To call variants with high confidence, Mutect2 (v4.2) and Lofreq (v2.1.5) were run for the detection of SNVs with candidate regions. In parallel, Mutect2, and Scalpel (v0.5.4) were run for the detection of Indels with candidate regions. The variants that overlap in the three algorithms would be considered true SNVs or Indels. In the variants calling, we used EGFP+ data as a control to identify the mutations that appeared in EGFP- data for each pair of samples. Furthermore, all identified and overlapped SNVs and Indels were confirmed by manual realignment, and variants locus in repeat sequence array would be removed.

For RNA-seq analysis, the total RNA from EGFP+/EGFP- cells was extracted by using the RNAeasy kit (Qaigen). RNA-seq was then performed in illumine platform, and raw data was trimmed and mapped by Hisat2 (v2.1.0), sorted by samtools (v1.10), and visualized by IGViewer (v2.12.3).

### Statistical analysis

R version 4.2.1 (https://www.r-project.org) was used to conduct all the statistical analyses in this study. All tests conducted were two-sided, and the significant difference was considered at *P* < 0.05.

### Supplementary Information

**Additional file 1: Supplemental Fig. 1.** Ovalbumin-EGFP fusion protein structures predicted by alphafold2 software. A. Structure of ovalbumin-EGFP fusion proteins with GS linker. B. Structure of ovalbumin-EGFP fusion proteins with (GS)_3_ linker. A. Structure of ovalbumin-EGFP fusion proteins with 32aa linker. A. Structure of ovalbumin-EGFP fusion proteins with (EAAAK)_5_ linker. **Supplemental Fig. 2.** Plasmids used in OVAL gene modified or CAG-EGFP chicken. A. Structure of CRISPR/Cas9 and donor plasmid used in ovalbumin locus site-specific gene integration. B. PiggayBac plasmid used in CAG-EGFP transgene chicken. **Supplemental Fig. 3.** EGFP fluorescence detection of PGCs derived from CAG-EGFP chicken. **Supplemental Fig. 4.** mCherry fluorescence detection of eggs from OVAL-E3-EGFP chicken. **Supplemental Fig. 5.** Immune fluorescence (IF) analysis of oviduct tissues from WT three-yellow chicken, CAG-EGFP chicken, and OVAL-E3-EGFP chicken. A. Ovalbumin antibody IF analysis of oviducts from WT three-yellow chicken, CAG-EGFP chicken, and OVAL-E3-EGFP chicken. B. EGFP antibody IF analysis of chicken oviduct from OVAL-E3-EGFP chicken. **Supplemental Fig. 6.** EGFP immune fluorescence analysis of oviduct tissues from wild type three-yellow chicken and CAG-EGFP chicken. **Supplemental Fig. 7.** FACS of EGFP+/EGFP- cells from OVAL-E3-EGFP chicken oviducts.

**Additional file 2: Supplemental Table 1.** Germline transmission of OVAL-EGFP F0 chimera. **Supplemental Table 2.** Size and volume data of eggs from wild-type and OVAL-E_3_-EGFP chicken. **Supplemental Table 3.** off-target effects detection in ovalbumin targeting chicken DNA (This data is a large list and thus list in another individual file.)

## Data Availability

All the sequencing data were deposited in the NCBI Sequence Read Archive (SRA) under project accession no. PRJNA903759.
