# Supplementary material for: An ovalbumin fusion strategy to increase recombinant protein secretion in chicken eggs
Source: J Biol Eng. 2024 Jan 11;18:5. doi: 10.1186/s13036-023-00390-4 (PMC10785457; doi:10.1186/s13036-023-00390-4)
Supplement: Supplementary file 1 — Additional file 1: Supplemental Fig. 1. Ovalbumin-EGFP fusion protein structures predicted by alphafold2 software. A. Structure of ovalbumin-EGFP fusion proteins with GS linker. B. Structure of ovalbumin-EGFP fusion proteins with (GS)3 linker. A. Structure of ovalbumin-EGFP fusion proteins with 32aa linker. A. Structure of ovalbumin-EGFP fusion proteins with (EAAAK)5 linker. Supplemental Fig. 2. Plasmids used in OVAL gene modified or CAG-EGFP chicken. A. Structure of CRISPR/Cas9 and donor plasmid used in ovalbumin locus site-specific gene integration. B. PiggayBac plasmid used in CAG-EGFP transgene chicken. Supplemental Fig. 3. EGFP fluorescence detection of PGCs derived from CAG-EGFP chicken. Supplemental Fig. 4. mCherry fluorescence detection of eggs from OVAL-E3-EGFP chicken. Supplemental Fig. 5. Immune fluorescence (IF) analysis of oviduct tissues from WT three-yellow chicken, CAG-EGFP chicken, and OVAL-E3-EGFP chicken. A. Ovalbumin antibody IF analysis of oviducts from WT three-yellow chicken, CAG-EGFP chicken, and OVAL-E3-EGFP chicken. B. EGFP antibody IF analysis of chicken oviduct from OVAL-E3-EGFP chicken. Supplemental Fig. 6. EGFP immune fluorescence analysis of oviduct tissues from wild type three-yellow chicken and CAG-EGFP chicken. Supplemental Fig. 7. FACS of EGFP+/EGFP- cells from OVAL-E3-EGFP chicken oviducts. [file 13036_2023_390_MOESM1_ESM.docx]

**Supplemental Figures**


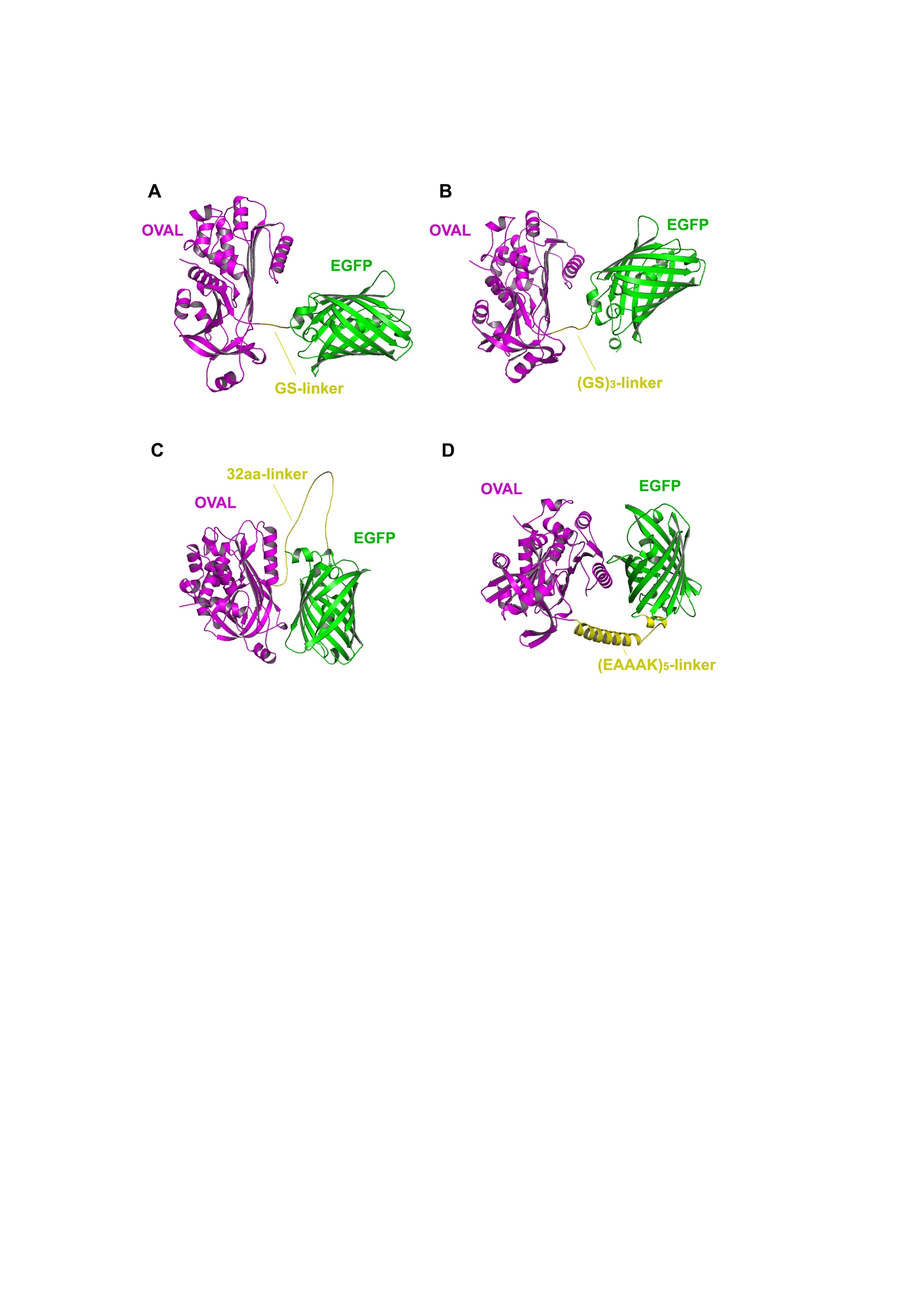


**Supplemental Figure 1. Ovalbumin-EGFP fusion protein structures predicted by alphafold2 software.** A. Structure of ovalbumin-EGFP fusion proteins with GS linker. B. Structure of ovalbumin-EGFP fusion proteins with (GS)_3_ linker. A. Structure of ovalbumin-EGFP fusion proteins with 32aa linker. A. Structure of ovalbumin-EGFP fusion proteins with (EAAAK)_5_ linker.


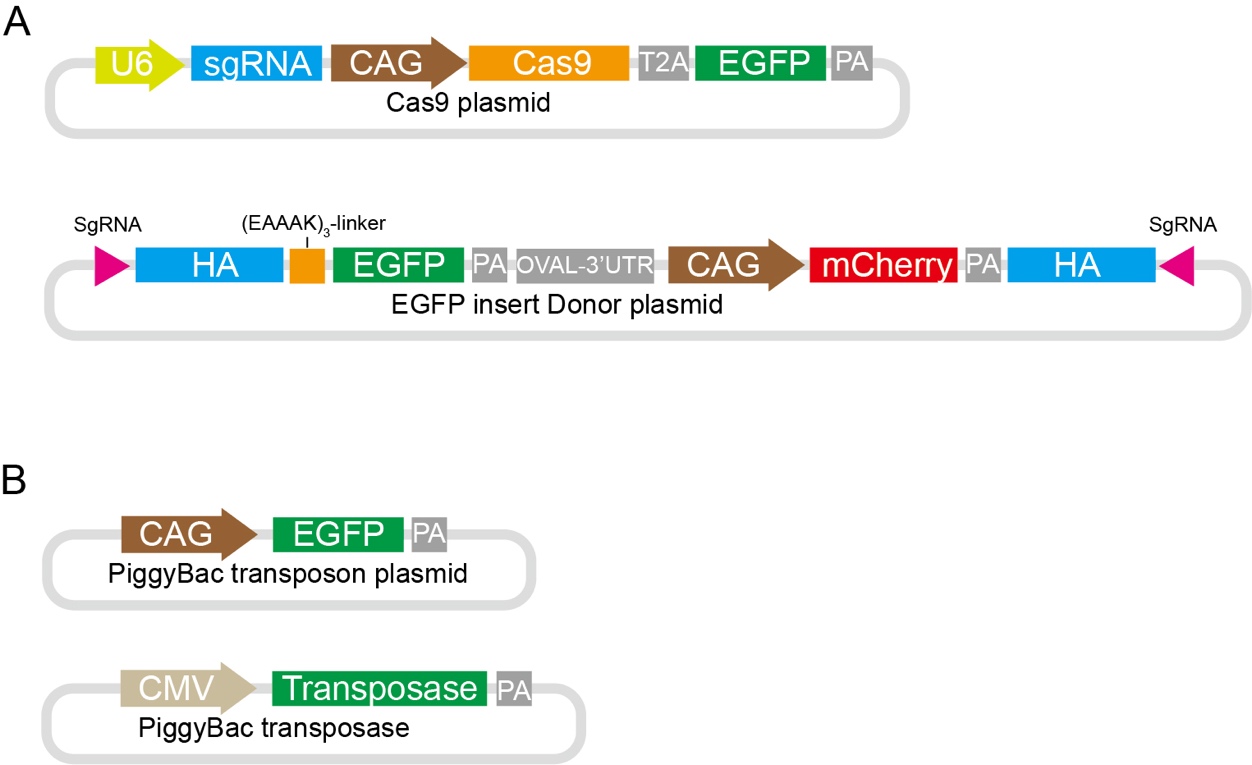


**Supplemental Figure 2. Plasmids used in OVAL gene modified or CAG-EGFP chicken.** A. Structure of CRISPR/Cas9 and donor plasmid used in ovalbumin locus site-specific gene integration. B. PiggayBac plasmid used in CAG-EGFP transgene chicken.


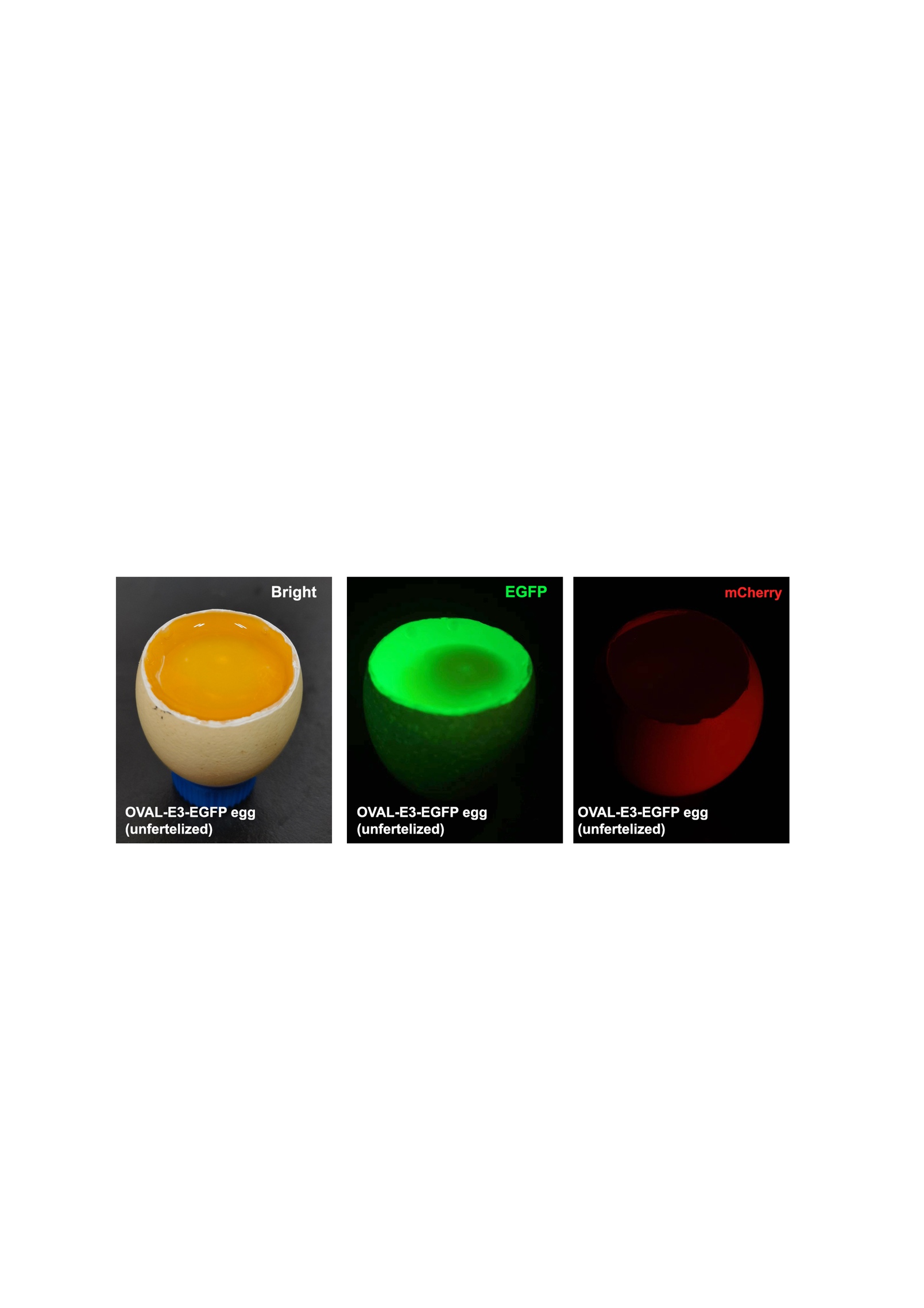


**Supplemental Figure 3. mCherry fluorescence detection of eggs from OVAL-E3-EGFP chicken.**


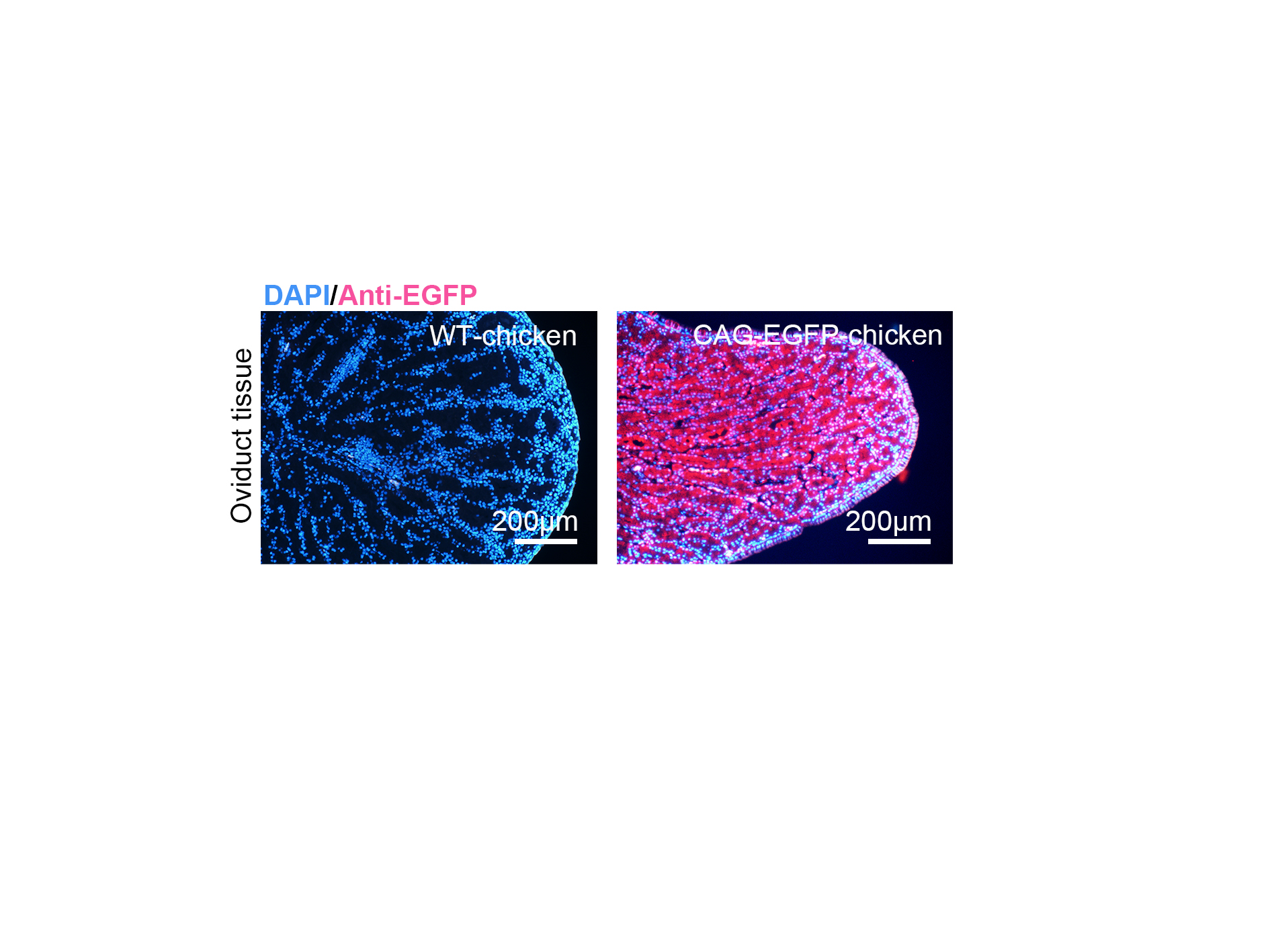


**Supplemental Figure 4. EGFP immune fluorescence analysis of oviduct tissues from wild type three-yellow chicken and CAG-EGFP chicken.**


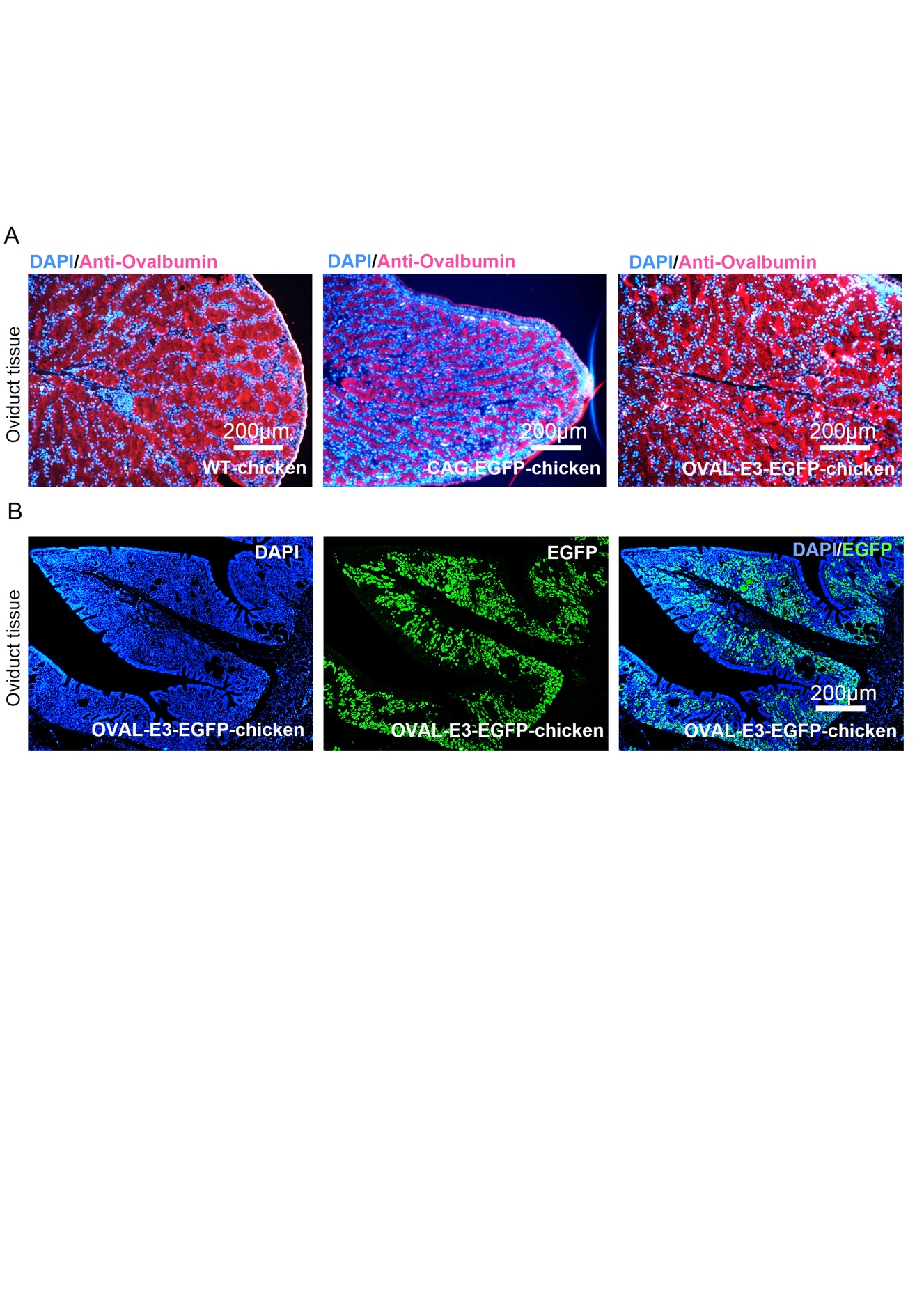


­­

**Supplemental Figure 5. Immune fluorescence (IF) analysis of oviduct tissues from WT three-yellow chicken, CAG-EGFP chicken, and OVAL-E3-EGFP chicken.** A. Ovalbumin antibody IF analysis of oviducts from WT three-yellow chicken, CAG-EGFP chicken, and OVAL-E3-EGFP chicken. B. EGFP antibody IF analysis of chicken oviduct from OVAL-E3-EGFP chciken.


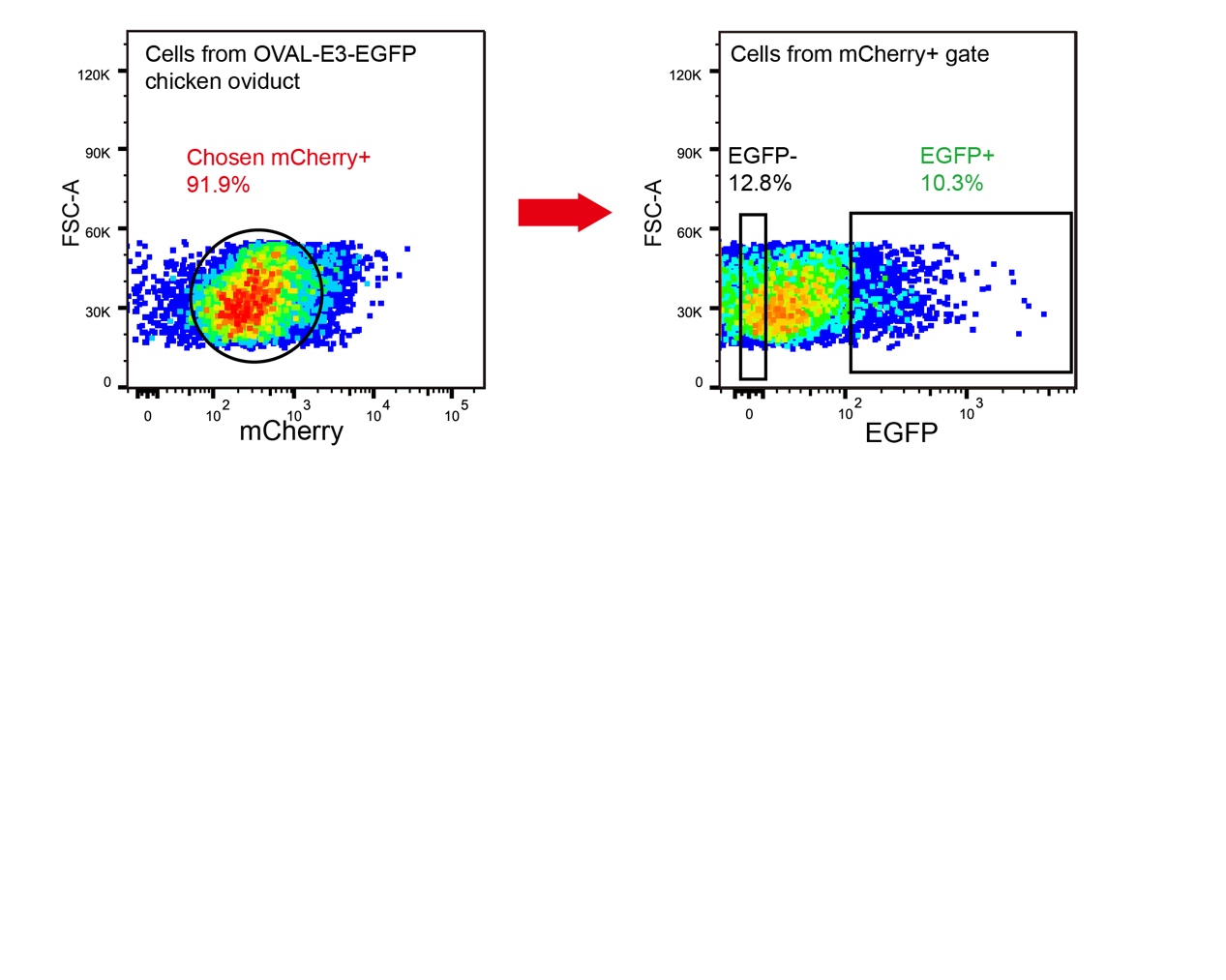


**Supplemental Figure 6. FACS of EGFP+/EGFP- cells from OVAL-E3-EGFP chicken oviducts.**


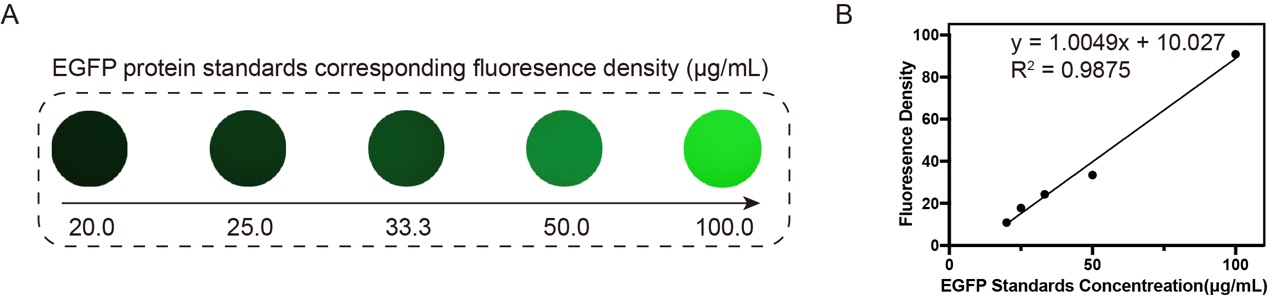


**Supplemental Figure 7. The establishment of EGFP protein standard curve.** A. florescence density of EGFP protein standard samples with different diluted concentration. B. The standard curve established based on the florescence density of EGFP standards.
